# Stochastic heuristics for decisions under risk and uncertainty

**DOI:** 10.3389/fpsyg.2024.1438581

**Published:** 2024-08-06

**Authors:** Leonidas Spiliopoulos, Ralph Hertwig

**Affiliations:** Center for Adaptive Rationality, Max Planck Institute for Human Development, Berlin, Germany

**Keywords:** errors, decision making under risk and uncertainty, model comparison, stochastic heuristics, bounded rationality

## Abstract

Models of heuristics are often predicated on the desideratum that they should possess no free parameters. As a result, heuristic implementations are usually deterministic and do not allow for any choice errors, as the latter would require a parameter to regulate the magnitude of errors. We discuss the implications of this in light of research that highlights the evidence supporting stochastic choice and its dependence on preferential strength. We argue that, in principle, the existing models of deterministic heuristics should, and can, be quite easily modified to stochastic counterparts through the addition of an error mechanism. This requires a single free parameter in the error mechanism, whilst otherwise retaining the parameter-free cognitive processes in the deterministic component of existing heuristics. We present various types of error mechanisms applicable to heuristics and discuss their comparative virtues and drawbacks, paying particular attention to their impact on model comparisons between heuristics and parameter-rich models.

## 1 Introduction

Heuristics, though there are many varying definitions of them, viewpoints (cf. Gigerenzer and Goldstein, 1996; Kahneman and Tversky, 1996) and different classes (Mousavi and Gigerenzer, 2017), are typically defined as models with clearly spelled-out cognitive processes. Their aim is to describe and approximate the actual processes as opposed to as-if models of behavioral outcomes, such as optimization theories (e.g., Bayesian decision theory, expected utility maximization). Another aspect of models of heuristics is that they eschew complex calculations that overtax human abilities and they ignore some of the available information (Gigerenzer and Gaissmaier, 2011), yet often still may manage to outperform significantly more complex models (e.g., Gigerenzer and Brighton, 2009; Katsikopoulos et al., 2010). Beyond the evidence from the lab, heuristics often perform very well in the field (Şimşek, 2013; Katsikopoulos et al., 2021) including the business world as even CEOs rely on heuristics to navigate exceptional uncertainty (e.g., see the overview in Mousavi and Gigerenzer, 2014). Another feature of models of heuristics is that they are usually constructed without free parameters to be estimated from data; in essence, they are deterministic models. This is particularly true of fast and frugal or ecologically rational heuristics (see Gigerenzer et al., 1999, 2011; Todd et al., 2012, and a comparative discussion of how ecological rationality is considered in economics and psychology; Mousavi and Kheirandish, 2014). This contrasts the majority of choice and inference models that include free parameters (e.g., expected utility model, cumulative prospect theory, and drift-diffusion models). There are several reasons for eschewing free parameters, perhaps the most important one is that they risk to unduly increase the flexibility of a model, thereby accounting for many different data patterns including noise. If the data is noisy or the training data limited, flexible free-parameter models are vulnerable to over-fitting to the noise in the data, thereby resulting in worse out of sample predictive performance than models without free parameters (e.g., see Gigerenzer and Brighton, 2009).

Fully deterministic models of heuristics, however, also exact a cost. They make it difficult to model both between-participants and within-participant heterogeneity. People are known to invoke different cognitive process across one and the same task, and the same person may switch to different processes even within the same class of task, depending on contextual factors such as time pressure (e.g, Svenson and Maule, 1993; Spiliopoulos and Ortmann, 2018), incentives (e.g., Payne et al., 1997) or task characteristics (e.g., choice difficult; see Brandstätter et al., 2008). Flexibility in the use of heuristics, both across individuals and within an individual and across environments, is inherent to the notion of the adaptive decision maker (Payne et al., 1997), and the adaptive tool box of heuristics as the basis of adaptive behavior (Gigerenzer et al., 2011). Given the unavoidable between-participants heterogeneity and the theoretically postulated within-person flexibility across properties of the choice environment, how can deterministic models of heuristics be allowed some flexibility without falling into the trap of too much flexibility? In the interest of full disclosure, we are sympathetic to a parameter-free approach (e.g., Spiliopoulos and Hertwig, 2020). Even if only to explore how much predictive power deterministic models have. Nevertheless, in this manuscript we are predominantly interested in a type of stochasticity arising within a person and within a task from errors in cognitive processes, to which no cognitive model, not even heuristics, are immune.

The desideratum of models of heuristics to avoid free parameters has consequently led to the majority of heuristics being implemented as deterministic, for instance, choosing a single option with certainty. This is because allowing for stochastic choice through errors inadvertently requires a free parameter to modulate the magnitude of errors. It is not clear how to avoid this without the arbitrary choice of such an error value that would not be fitted to data and likely not representative of its true value. We argue that the science of heuristics needs to seriously consider the pros and cons of the existing strict adherence to no free parameters (see also, Ortmann and Spiliopoulos, 2023), and allowing flexibility in perhaps the most important place, namely, with respect to stochastic choice arising from errors.

We will argue that transitioning toward some flexibility offers several opportunities, including methodological improvements particularly in model comparisons. Furthermore, modifying heuristics so that they are able to predict a strength of preference over options, rather than a deterministic choice will increase the empirical content of heuristics and make them more falsifiable. We will discuss how this will level the playing field when comparing flexible models with free parameters against models of heuristics, as current practices involving deterministic models of the latter may be problematic. Given the strong procedural foundations of heuristics, one can consider errors in a more principled and structured way than is possible with as-if behavioral models. This is because the clearly defined and transparent processes in heuristics suggest how errors come about and constrain the error distributions, whereas with as-if models it is harder to arrive at a priori reasonable constraints.

Let us briefly define some terms that are used throughout. We will think of models as consisting of two components: the first one is indispensable and is the *core deterministic* component; the second one represents an *error-mechanism* (or stochastic) component and is often referred to as a choice rule. A *deterministic* model always chooses one of the available options with certainty (i.e., probability 1.0), wholly rejecting all other options (i.e., probability zero)—this choice distribution is *discrete*. *Continuous* choice distributions, in contrast, imply that at least one choice is made with a probability greater than zero and less than one. As mentioned before, most implementations of models of heuristics are deterministic; in this manuscript we will refer to them as deterministic models of heuristics, as opposed to stochastic models of heuristics that permit continuous choice distributions. Finally, a *flexible* behavioral model is one that has free parameters in the core component that are typically estimated from data. That is, according to our terminology, a model without free parameters in the core but with an error mechanism that includes one or more free parameters, is not a flexible model. For our purposes, such models are *stochastic* models of heuristics.

Models with free parameters are inferred from data using an *estimation technique*, which requires the specification of a *loss function*, e.g., mean-squared-deviation or a likelihood function. To avoid issues of flexible models over-fitting empirical data, we only consider model performance out-of-sample as derived from a performance metric. Such metrics may also be *discrete* (as the prediction is an extreme choice of 0 or 1) or continuous—note, discreteness or continuity of the metric refers to each individual choice, not to the average metric applied over many choices. For example, consider a metric such as the percentage correct predictions that is the average of the values of 0 (if a choice was not correctly predicted) or 1 (if it was). At the individual choice level, this is discrete, but the final metric constructed from the average of these values is continuous. We refer to this metric as discrete, to differentiate it from another metric that may make probabilistic (continuous) individual predictions, say that one option is chosen with probability 0.8 and the other 0.2, but which again when averaging over choice predictions would also return a final continuous measure.

Models consist of various processes ranging from information search to information integration and each process may be prone to error. We refer to the final process that leads to a choice, as the *valuation stage* and it typically involves the comparison of a set of values, one for each of the options available—errors that happen during this stage are referred to as *valuation errors*. Earlier processes will also often involve numerical comparisons, but typically these numbers would not represent a final valuation—these are coined *procedural* errors.

The manuscript is structured as follows. First, we briefly overview the overwhelming evidence that points to significant stochasticity in choices and how it relates to a decision maker's strength of preference over the available options. We then proceed with methodological arguments for stochastic models of heuristics, highlighting some of the problems that may arise if analyzes are based solely on deterministic variants. In a subsection, we will deal specifically with issues that may arise in model comparisons between flexible models and heuristics if the latter are not modeled as stochastic. Having laid the foundations for why we consider stochastic models of heuristics to be important, we lay out a classification of applicable error mechanisms. The ensuing comparative discussion about the advantages and shortcomings of each will allow us to make practical recommendations about their implementation. We will illustrate these by presenting possible stochastic variants of the popular maximin heuristic. Our emphasis throughout is on models of choice heuristics under risk and uncertainty. Yet, our arguments are easily translated to models of heuristics in general. Last but not least, let also emphasize that the question of whether, and how, to incorporate flexibility in deterministic models of heuristic models has been discussed before (see Rieskamp, 2008; Schulze et al., 2021); we will discuss this work below.

## 2 Arguments for stochastic heuristics

### 2.1 Decision making is stochastic

There is strong evidence in favor of the proposition that choice is inherently stochastic—see Rieskamp (2008) for a detailed discussion. In choice under risk, participants presented with identical lotteries under risk often make different choices when repeatedly responding to them (Hey, 2001; Mata et al., 2018). Some choice theories such as cumulative prospect theory are often amended by adding a choice rule that accommodates such errors. The underlying cognitive processes that may underlie choice behavior are also error prone or noisy, for example, memory retrieval and attention. Consequently, some theories are constructed to be inherently stochastic by nature, such as evidence accumulation models of behavior (e.g., Ratcliff, 1988; Busemeyer and Townsend, 1993; Usher and McClelland, 2001), where the accumulation process itself is stochastic, but the final step of hitting a decision threshold is error free (i.e., the choice corresponding to the threshold is chosen with certainty).

Choice stochasticity from the viewpoint of an observer (such as researchers) may also be attributed to other causes. Even if a deterministic decision-maker were to exist, to an observer that does not have access to the exact states of all variables entering the decision processes, choices will appear stochastic due to the (unobservable) latent variables. This is analogous to the example of a die roll being essentially deterministic, yet appearing as stochastic to observers that do not have access to the exact initial conditions and physical values. The argument for extending choice models to be stochastic is therefore not just one of modeling realism (due to internal noisy cognitive processes), but is also related to the methodology of model estimation: How unobservable latent variables are accounted for, even indirectly, is important.

Ultimately, the research goal may dictate whether adding an error mechanism is desirable or not. If it is solely for prediction, then a deterministic heuristic with no free parameters may be preferable and adequate. Of course, the allure of deterministic models of heuristics is that they are powerful exactly because no data is needed for them to make behavioral predictions. A caveat is that if there is heterogeneity among decision makers or a decision maker flexibly uses different heuristics, then one would need empirical data to obtain an estimate of the proportional use of different heuristics. On the other hand, for robust model comparisons we believe it is advisable to accept the addition of free parameters in the error mechanism, whilst retaining the hallmark of models of heuristics—a deterministic and parameter-free model core. In model comparisons, ignoring errors risks being problematic as the models are essentially misspecified.

### 2.2 Preferential strength affects choice consistency

Choice consistency in a wide variety of tasks is a monotonically increasing function of the (absolute) relative strength of preference of an option over the remaining options. That is, errors are increasingly more likely and more substantial when options are relatively similar in their valuations. At a cognitive level, this can be understood in terms of just-noticeable differences or signal detection theory. Error-mechanisms and choice rules have a long history in cognitive psychology (Thurstone, 1927; Mosteller and Nogee, 1951; Luce, 1959) and economics (McFadden, 2001). The choice rules most often used in the literature are based on exactly this monotonicity assumption, e.g., logit and probit choice rules. In drift-diffusion models the magnitude of the drift is derived from the evidence in favor of each option, and the higher the magnitude of the drift rate, the more extreme the choice predictions and, correspondingly, the higher the choice consistency. Independent of specific parametric forms, empirical evidence for a strong monotonic relationship between consistency and preferential strength in choice under risk and intertemporal choice is presented in Alós-Ferrer and Garagnani (2021, 2022). Further indirect evidence of the important role of preferential strength is evident from the finding that response times are typically longer the closer the valuation of the options is (Moffatt, 2005; Chabris et al., 2009; Spiliopoulos, 2018; Spiliopoulos and Ortmann, 2018; Alós-Ferrer and Garagnani, 2022).

Flexible models have been implemented with error-mechanisms more often than models of heuristics for several reasons. In flexible models, options typically receive some absolute value in the final valuation stage. From here it was but a small step to consider preferential strength and how this may map to continuous choice probabilities. An example of this is the expected utility of each prospect in a pair of lotteries, which is typically translated into a probability distribution over options using an error mechanism that is a function of option valuations. Perhaps the hesitation in considering stochastic models of heuristics is the concern that it requires one of two things: (a) complex parametric forms to calculate continuously-valued option valuations in combination with a choice rule and/or (b) multiplicative integration of probabilities and outcome values in contrast to the simpler comparative and logical operations found in models of heuristics (e.g., comparison of magnitudes). We will show later that this concern may be unwarranted in many cases, as option valuations and preferential strength can be trivially inferred from existing heuristics for choices under risk and uncertainty, without changing their deterministic parameter-free core and the assumption of simple processes.

### 2.3 Methodological arguments

Heuristics have been presented as procedural models of behavior that are more realistic than their parameter-rich as-if adversaries. Scholars advocating for models of heuristics have correctly, in our opinion, asserted that comparisons of heuristics and flexible models should be done on the basis of out-of-sample or cross-validation performance. The argument is that good performance by flexible models with many free parameters is illusive if their performance is estimated in sample. Ultimately, comparisons between the two types of models comes down to their out of sample performance on the same sets of tasks; however, the difference in their need for estimation may be problematic when it comes to such a model comparison. Can flexible stochastic models and deterministic models of heuristics be directly compared without unduly handicapping one or the other? We wish to draw attention to some issues with existing methods of comparing these models and suggest a viable alternative that may alleviate them—see related arguments about model comparisons in Spiliopoulos and Hertwig (2020).

The first issue concerns the fact that flexible models are usually implemented with an error mechanism, and are therefore stochastic, admitting continuous-valued predictions (on the probability scale) derived from valuations, whereas heuristics are deterministic, admitting discrete-valued predictions only. How is this difference typically reconciled in the literature? For a direct comparison, both models must be scored according to the same performance metric leading to three possible solutions:

1. Use a discrete performance metric and convert the continuous-valued predictions of a stochastic flexible model to discrete predictions to be compared against a discrete heuristic.
2. Use a discrete performance metric and deterministic flexible models and heuristics, so that the above conversion need not to be made.
3. Use a continuous performance metric with both flexible models and heuristics implemented with a stochastic error mechanism.

We believe that the first two, which are predominantly used in the literature, may be problematic in various respects, and recommend the third option—let us explore the reasoning behind our assertion.

#### 2.3.1 Option 1

This option is problematic because of the mismatch between the *continuous* loss function necessitated by the estimation of the stochastic flexible model and the subsequent application of a *discrete* performance metric. Converting a stochastic prediction to a deterministic one is usually achieved by assuming that the option with the highest predicted likelihood is chosen with probability 1 and the other options with probability 0. Having done this conversion, both flexible models and heuristics can be compared using the percentage of correct choice metric, ignoring any probabilistic information that existed in the flexible models (and by extension in the choice data). This is clearly inefficient and may have put flexible models at a relative disadvantage to heuristics, as they are estimated using a procedure with a different goal or metric than the one that their comparison to heuristics is based on, possibly leading to poorer performance than would otherwise be the case. This occurs because the nature of the loss function determines the parameter estimates, which in turn affect the choice predictions. Consider how the continuous L2 and log-likelihood loss functions penalize errors during estimation. Since the penalty for the error is a continuous function of the error magnitude, the errors between a continuous-valued prediction of 0.49 and 0.51 are very similar in value (assuming two options). Now consider the discrete performance metric, which requires that those two predictions are discretized to values of 0 and 1, respectively. The errors are now diametrically opposed, one prediction has an error of 1 and the other 0. In general, the true (continuous) magnitude of the error is irrelevant under discrete loss functions (and performance metrics) as long as it is on the same side of 0.5. Under continuous loss functions, larger errors are always penalized more, whereas under discrete loss functions errors are penalized more only when the threshold prediction of 0.5 is crossed, jumping discontinuously at this point. These significant differences substantially influence the estimation procedure and the resulting parameter estimates, possibly leading to worse predictive performance than if the parameters were fitted with a loss function identical to the performance metric.

There are important drawbacks to using a discrete performance metric. As the link between preferential strength and choice probabilities is severed by this metric, deterministic heuristics may be placed at a relative advantage to flexible models, as the advantage of the latter in accounting for preferential strength is ignored. Also, as discrete model predictions are less precise than continuous predictions, this makes models less identifiable or distinguishable, less falsifiable and more prone to model mimicry, thereby hampering efficient model comparison.

The topic of model mimicry has received increasing attention in the methodological literature, particularly with respect to its impact on model comparisons. Sets of flexible models can often exhibit significant model mimicry exactly because if endowed with numerous free parameters they can fit almost any data. Deceivingly, significant model mimicry can be found even across models that appear to have very different foundations and non-linear parametric forms, if there are enough parameters to interact with each other. Recall von Neumann's quip to Fermi, that “With four parameters I can fit an elephant, and with five I can make him wiggle his trunk.” Concerning model comparisons between flexible models such as cumulative prospect theory and choice heuristics, significant model mimicry has been found even for such different models (Brandstätter et al., 2006, Table 5; Pachur et al., 2013). One perspective is that this is a feature of heuristics, since it implies that they can have approximately the same predictive performance as flexible models with much simpler functional form and a lack of free parameters. We agree, but wish to point out that these model mimicry comparisons have typically been performed on discrete prediction metrics, which necessary preclude any informativeness that may be derived from strength-of-preference (and by extension, from stochastic variants of said models). An exception is the model recovery analysis by Pachur et al. (2013), who showed how Cumulative Prospect Theory can mimic a wide variety of heuristics with very difference processes, through the flexibility afforded by the probability weighting function parameters. In this study, CPT and heuristics were rendered stochastic through a fixed error mechanism, and the success of model recovery was determined for varying levels of errors. We suspect that comparing models not on a discrete metric but on a continuous metric (and stochastic variants of the models) such as choice probability after the introduction of a choice rule, will reveal less model mimicry than previously observed. This is a corollary to the argument that stochastic models are more falsifiable than their deterministic counterparts due to their more precise predictions covering the full probability range.

How large can this difference be theoretically? Consider the following example presented in Table 1. Let us take a simple case where decision-makers are presented with two different pairs of lotteries, each with two prospects A and B. The lotteries are repeated 100 times each, so that consistency and stochasticity can be revealed. If a discrete performance metric is used, then perfect model mimicry would be observed under the following conditions. Consider the empirical choice data first (the last column in the table), and let us assume that A was chosen in Lottery pair 1 99% of the time and 51% of the time in Lottery pair 2. Note that this would likely be the case if the two prospects in Pair 1 had valuations that were very different, leading to few errors. In Pair 2, in contrast, the two valuations of both prospect result in very similar values, leading to many errors and a near 50–50 choice proportion.

**Table 1:** A comparison of model mimicry and performance between deterministic and stochastic models.

	**Model predictions**				**Choice data**
	**Deterministic**		**Stochastic**		
**Task**	**Model 1**	**Model 2**	**Model 1**	**Model 2**	
#1	0.99	0.99	0.99	0.51	0.99
#2	0.51	0.51	0.51	0.99	0.51

Now consider deterministic versions of two models, both of which predict prospect A as being chosen with certainty. In this case, both models would have the same predictive accuracy of 99 and 51% for Pairs 1 and 2, respectively, implying perfect model mimicry—see the first two columns in the table. That is, the two models' predictions are perfectly positively correlated across the lotteries. Suppose that the stochastic version of Model 1 predicts *p*(*A*) = 0.51 for Lottery 1 and *p*(*A*) = 0.99 for Lottery 2, whereas these values are flipped for Model 2. This is entirely consistent with the numbers used for the deterministic models above, as long as the stochastic versions both predict a choice probability for Prospect A greater than 0.5 for both lotteries—this would imply choosing A with certainty under a discrete metric. The predictive accuracy of Model 1 is now 99 and 51% for lotteries 1 and 2, whereas that of Model 2 is 51 and 91%, respectively. Examining the correlation between the two models and across the lottery predictions reveals that they are perfectly *negatively* correlated, in contrast to the perfect *positive* correlation between the deterministic models. Consequently, model mimicry was significantly over-estimated in the latter case. It is clear from the empirical (true) choice data, that Model 1 is preferable, however this can only be concluded by comparing the stochastic model variants with a continuous metric, not by their deterministic models.

#### 2.3.2 Option 2

The second option is also problematic for numerous reasons—note, the arguments made above regarding discrete performance metrics continue to hold in this case. The strong empirical evidence that choice behavior is generally stochastic implies that deterministic models are strongly misspecified during estimation. Consequently, inferring that one deterministic model or the other has been invalidated by a model comparison is wrought with difficulty, as deviations of model predictions from the empirical data cannot necessarily be attributed to the core deterministic model being wrong, but may be due to the lack of an error mechanism. This is particularly problematic for studies that employ the axiomatic approach to invalidating a model (or that include specifically designed tasks to stress-test axioms). For example, deterministic EUT assumes transitivity of choices, which is not supported by the empirical data as we often observe violations. However, this does not preclude the deterministic component of EUT being correct, and that any violations of the transitivity axiom arise solely due to errors. Similarly, violations of stochastic dominance may arise either in the core deterministic model or from an error component (or both).

How problematic can this become? We perform a simple recovery simulation where the true choices or data are generated using a stochastic model and perform a model comparison analysis using deterministic models. If the deterministic model recovered matches the core deterministic component of the true stochastic choice model, then we deem this as a correct recovery. For example, if we generate the choice data using a stochastic Expected Value (EV) model, do we conclude often enough that the core component was the EV model even when our model comparison assumes a deterministic EV model and other competing deterministic heuristics?

We implement the simulation using heuristics that are often used in the choice under risk literature (Thorngate, 1980; Payne et al., 1988; Hertwig et al., 2019). The set of models consists of the Expected Value model, which uses all information (probabilities and outcomes), and the following heuristics that either ignore or process probability information in a non-multiplicative way: Maximax, Maximin, Least likely (LL), Most likely (ML), Equiprobable (EQ), Probable (Prob), and the Priority heuristic.^1^ Assume that a decision-maker uses the same stochastic heuristic to make choices in *N* choice tasks or lotteries. Given the practical limitations of experiments, setting *N* = 50 is a reasonable assumption. Two prospects for each lottery are randomly drawn in the following fashion. Both prospects consist of two outcomes each and probabilities are drawn from a uniform distribution drawn over [0, 1] and the outcomes are drawn from a uniform distribution over [0, 100].

We examine the case of two different stochastic models as the true choice models, EV and Maximin. Stochasticity is modeled as noise in the outcome values. This is a simplification, of course, as noise could also affect probabilities. However, since many heuristics ignore probabilities, but not outcome information, we settled on the latter. We vary the degree of noise or stochasticity in the data generation process by adding errors to each outcome value that are normally distributed with mean 0 and standard deviation equal to 5, 10, 20, 50, or 100.

Summarizing, for each true choice model and associated noise level, we calculate the recovery rate for each of the eight decision models under investigation. Model comparison and recovery is based on the following performance metric: The best performing model that we infer is used by the decision-maker is the one with the highest percentage of correct predictions across the *N* tasks. The choice data generation and model prediction is simulated 10,000 times, and the recovery rate is defined as the percentage of those simulations for which the correct model was inferred. If the outcome noise is zero, then the recovery rate will necessarily be 100% as the stochastic and deterministic versions of a model are identical.

Tables 2, 3 present the recovery rates for each of the two true models (stochastic EV and Maximin, respectively) for each noise level. If the true model is stochastic EV, for low noise (σ = 5) the recovery rate is very high (97%); however, as the level of noise increases recovery falls significantly to 70% for a noise level of 20. At high levels of noise (50 and 100), the recovery rate falls to 41 and 26%, indicating a significant failure in recovering the true model by deterministic models of choice behavior. When recovery fails, the most commonly inferred (incorrect) models are ML and Probable. This constitutes a significant failure as they are in principle quite different models from EV, ignoring some of a prospect's events and not fully utilizing probabilistic information. For high levels of noise (50 and 100), even more parsimonious heuristics may be incorrectly inferred as the true model, in particular ones that completely ignore probabilistic information (i.e., Maximax and Maximin).

**Table 2:** Recovery rates (%) if the true model is stochastic EV.

**Noise σ**	**EV**	**Maximax**	**Maximin**	**LL**	**ML**	**Eq**	**Prob**	**Priority**
5	97	0	0	0	6	2	6	0
10	89	1	1	0	15	7	15	0
20	70	5	4	0	26	15	26	2
50	41	15	13	7	29	21	29	9
100	26	19	16	16	24	19	24	14

**Table 3:** Recovery rates (%) if the true model is stochastic maximin.

**Noise σ**	**EV**	**Maximax**	**Maximin**	**LL**	**ML**	**Equip**	**Prob**	**Priority**
5	0	0	86	0	0	3	0	26
10	3	0	71	0	0	11	0	38
20	10	1	50	0	3	26	3	38
50	19	15	28	2	11	31	11	28
100	18	22	22	10	16	24	16	24

Let us now turn to the case where a stochastic Maximin model generates choices. At the lowest level of noise (5), the recovery rate is 86%, falling to 71% for the next noise level (10), and only 50% for a noise level of 20. Compared to the case where the stochastic EV was the true model, recovery rates for stochastic Maximin are generally worse at the corresponding noise levels, and drop more quickly even for intermediate noise levels. The most common wrongly inferred model is the Priority heuristic, which is understandable as the latter shares a very similar first step in the lexicographic decision tree. The recovery rate of 50% at a noise level of 20 is quite poor, and even more problematic is the fact that some of the wrongly inferred models are significantly different to Maximin. The second most commonly inferred wrong model is Equiprobable, followed by EV. Equiprobable is a significantly different heuristic to Maximin in principle, as it examines all outcomes rather than just the minimum outcomes in each prospect. Even more concerning is that in the presence of noise the EV model may be inferred as the true model, even though it is the antithesis of the Maximin heuristic. Our recovery simulations—while relatively simple abstractions of more complex model comparisons—have shown that there is cause for concern regarding the accuracy of model inference when heuristics are incorrectly assumed to be deterministic instead of stochastic. Further simulations seem warranted to investigate the accuracy of model recovery: (a) in a broader set of tasks where lotteries are sampled differently, (b) for a broader range of models, including flexible models such as CPT, (c) and for various categories of error mechanisms as defined in the next section.

Another issue with this option is that deterministic models necessitate discrete loss functions and performance metrics. This leads to a loss in the informational content of the empirical data, which by its nature is stochastic. Finally, using a discrete error function to estimate a flexible model (whether deterministic or stochastic) is extremely problematic due to key properties relating to the behavior of the loss function with respect to the estimation technique. For example, estimation based on minimizing the percentage of correct predictions is generally avoided as there is no guarantee of a unique solution in the parameter values due to the discreteness and lack of continuity of this loss function, i.e., different parameter values can lead to the same percentage of correct predictions, and it is not guaranteed that the estimation algorithm will converge to a global rather than local optimum. Since it is clearly desirable to estimate flexible models using continuous loss functions and to use an identical loss function and performance metric, this leaves only the next option as a viable candidate.

#### 2.3.3 Option 3

This option in our opinion dominates the two previously discussed ones, yet to the best of our knowledge has not been extensively used in the literature for a wide range of flexible models and heuristics, only for a limited number of models in rare cases (e.g., Rieskamp, 2008). First, it deals with the misspecification issue as both types of models are implemented as stochastic and prone to errors. Secondly, the mismatch between the loss function and the performance metric can be eliminated for both types of models, by using a continuous error function with an identical performance metric. Unifying the loss function, estimation technique and performance metric for both models minimizes the auxiliary assumptions involved in any model comparison (in the spirit of the Duhem-Quine problem), lending further credibility to the comparison conclusions. The stochastic specifications will make the models more identifiable and falsifiable, by making more precise predictions on the continuous probability interval compared to discrete predictions with certainty, as argued above.

### 2.4 Discussion

To conclude, there are important reasons to consider stochastic models of heuristics and to compare flexible models and heuristics using continuous performance metrics, in contrast to the majority of studies that have used discrete metrics (e.g, Pachur et al., 2013). First, they are cognitively more realistic as choice is stochastic (or noisy) and dependent on preferential strength. Second, such models would allow for a more equitable comparison of heuristics versus flexible models by doing away with differences in auxiliary assumptions, using the full informational content of data and the more precise predictions of continuous choice probabilities.

Furthermore, stochastic models of heuristics will be more falsifiable than their deterministic counterparts, as they will be forced to also account for preferential strength to perform well. This is a crucial test for heuristics that has not been empirically conducted yet. It may lead to further innovation in the field if the existing models of heuristics are not found to predict preferential strength well.

It is conceivable that some deterministic models of heuristics that have been rejected as not predicting behavior well in past studies, may in fact have fallen prey to their lack of an error mechanism that could “explain” some deviant choices. Simply put, the misspecification of heuristics as deterministic may invalidate conclusions drawn from deterministic heuristic modeling comparisons, as we showed in our recovery simulation. To be fair, all models are misspecified, but given how elemental stochastic choice seems to be in every facet of human behavior, the omission of an error mechanism may be more important that other sources of misspecification, such as a parametric form that is not exactly faithful to its true form.

## 3 A classification of error mechanisms

We now classify various types of error mechanisms that are applicable to models of heuristics, and discuss their advantages and shortcomings. We will use the maximin heuristic as a case study of how to define a stochastic variant. It is well-known both in individual choice under risk and uncertainty, and also in strategic decision making, such as games where the choices of other influence one's own payoffs (see Spiliopoulos and Hertwig, 2020). The maximin heuristic recommends that the chosen prospect is the one that has the most attractive worst-possible outcome. This heuristic is non-probabilistic and as it only compares outcome values across prospects and is thus an instance of the class of fast-and-frugal heuristics.

### 3.1 Fixed (or independent) errors

Stochasticity arising from fixed errors is not conditional on any of the processes involved at arriving at a choice. Alternatively, they are sometimes referred to as naive errors, as they simply stipulate that the deterministically derived choice is mistakenly not chosen in ϵ% of choices. Thus, if two options are available, the stochastic model of a heuristic will predict the choice of the deterministic model of the heuristic 100−ϵ% of the time and the other choice ϵ%. If more than two options exist, then one must stipulate how the errors are spread to the other option. The most obvious choice that retains independence is to apportion the ϵ% of errors uniformly over the other options. A stochastic version of maximin would therefore choose the option with the best worst-case scenario 100−ϵ% of the time.

The advantage of fixed errors is that they are quite simple to implement and can be useful in cases where there may be multiple errors occurring prior to the final choice, but which would be too difficult to estimate and effectively identify during estimation. Thus, the cumulative effect of the errors during the decision processes will be estimated, with the cost that this distribution may not effectively capture the true error distribution. Note that fixed errors can be used even with models without a final valuation stage from which a strength of preference could be inferred.

A useful extension of fixed errors are *conditionally* fixed errors, where a fixed error parameter may be valued differently conditional on characteristics of the task. For example, errors may be more likely for more difficult tasks than for easier tasks. Returning to the example above, suppose a decision-maker must choose between two options in one case and four options in another—the probability of making an error is likely higher in the latter case than in the former. This could be modeled by allowing the value of the error rate ϵ to be conditional on the number of available options. Of course, this comes at the cost of additional free parameters to be estimated.

A disadvantage of this fixed error mechanism is that it is still not ideal when used in conjunction with a continuous performance metric. For all tasks the choice predictions take on only two possible values, ϵ and 1−ϵ, whereas a continuous metric can take on the full range of values between 0 and 1. This happens exactly because fixed errors are not conditional on preferential strength, which will typically vary across tasks allowing model predictions to take on a broader range of values instead of two discrete values. If the core model includes a valuation stage, then the next type of errors would be more desirable, as they allow the size of the error to be conditional on the measure of preferential strength derived from the valuations. This, in turn, would enable probabilistic predictions that are not constrained to just two values, ϵ and 1−ϵ.

### 3.2 Valuation errors

Valuation errors are perhaps the most commonly employed error-mechanisms for flexible models. Valuation based error mechanisms are conditional on the relative magnitude of the valuations, which can be interpreted as a strength of preference. This error mechanism is more sophisticated and realistic than a fixed error mechanism—recall the evidence we presented earlier about the link between preferential strength and errors (or consistency).

How could such a mechanism be implemented in a deterministic heuristic, which usually do not have an explicit valuation stage? Let us turn again to the maximin heuristic. A prospect *i* is defined by the *n* possible outcomes and associated probabilities *p*_*n*_. The maximin heuristic can be procedurally calculated in three steps:

1. Determine the minimum value in each option.
2. Compare the minimum values and find the option with the larger minimum value.
3. Choose this option.

The choice rule is based on the comparison between these two minimum values. Regardless of the magnitude of the differences between the minimum values, the heuristic uses an all-or-nothing rule in the final choice. What if a rule is used that depends on the difference between the two minimum values? That is, let us define the valuation of a prospect as its minimum outcome value, and interpret the difference in the two minimum values as defining the continuous strength-of-preference for one prospect over another. The higher the preferential strength, the more likely the prospect is to be chosen, meaning that choice probability is an increasing function of strength of preference. Consequently, a stochastic maximin heuristic could be defined as follows, where λ = ϵ^−1^ is the consistency parameter:


$$\begin{array}{l} p_{A}=f\left(\min X_{A},\min X_{B},\lambda \right)\\ \;\frac{\partial p_{A}}{\partial \left(\min X_{A}\right)>0}\\ \;\frac{\partial p_{A}}{\partial \left(\min X_{B}\right)<0} \end{array}$$

It is convenient to choose a parametric function *f* such that if the error parameter ϵ is zero the function will return the same prediction as the deterministic maximin heuristic. The advantage of this is that by estimating ϵ it is possible to actually ascertain *how* stochastic choice is, and it also includes the special case of the deterministic heuristic, if warranted by the data. An obvious candidate is the logit (or probit) function alluded to earlier, see Equation 1. As λ approaches infinity, the probability of choosing one of the prospects tends to 1 and the other to 0, i.e., identical to that made by deterministic maximin.


(1) $$\begin{array}{l} p_{A}=\frac{e^{\lambda \left(\min X_{A}\right)}}{e^{\lambda \left(\min X_{A}\right)}+e^{\lambda \left(\min X_{B}\right)}} \end{array}$$

The parametric form of the error mechanism *f* has been shown to be very important, affecting not only the estimated parameters as we have already discussed above, but also the predictive performance of decision models and the informativeness of model comparisons (Zilker, 2022). Using cumulative prospect theory as the core deterministic component, Zilker rigorously examined various forms of error mechanisms and concluded that independent or fixed errors are eclipsed by the informativeness of the valuation error mechanism that we propose. Schulze et al. (2021) also concluded in their probabilistic model of the social circle heuristic that valuation error mechanisms, logit and probit, significantly outperformed a fixed error mechanism. Stott (2006) performed an extensive comparison of all possible combinations of different parameterizations for cumulative prospect theory's probability weighting functions, value functions and error functions, also concluding that the logit outperformed fixed errors and was the best performing parameterization. Consequently, wherever possible, we recommend using a valuation error mechanism instead of a fixed error mechanism. Further research should be directed at considering the appropriate functional form of the error mechanism for models of heuristics because, as we discuss below, other more sophisticated alternatives exist.

### 3.3 Procedural errors

Procedural errors can occur at any processing level or step (with the exception of the valuation stage, which was covered above as a special case). A prerequisite for such an error mechanism is that a procedural model be clearly defined in terms of the requisite cognitive operations. An obvious approach for interpreting such a model is to define it in terms of elementary information processing units (EIPs) and to allow for an error in multiple, but ultimately, all of the EIPs. That is, errors occur at every level of information integration (and possibly search) instead of after integration is complete and a valuation returned. The resultant choice errors are caused by the propagation of the procedural errors throughout the model. For example, an error at an early EIP can interact with an error at a later EIP, thereby leading to a very rich distribution of final choice errors that may even be multimodal. This contrasts the unimodal error distributions associated with valuation error mechanisms as a result of the assumption that preferential strength is monotonically related to errors. While not the focus here, finding multi-modal (and a more discretized) rather than uni-modal (and continuous) error distributions may be a strong indication that the true core behavioral model is a heuristic rather than a flexible model. This conjecture may warrant further investigation as it may be a powerful way of identifying when heuristics are used by decision-makers.

Let us turn again to our Maximin example. The valuation error mechanism we implemented above assumed that the first step in Maximin—determining the minimum value in each option—was error free. A procedural error mechanism would introduce an error at this step. The procedural error that occurs in comparing outcomes within each option could be implemented as an independent error with fixed probability of occurring or as an error conditional on the difference between the compared values (e.g., the minimum and maximum outcomes in a two-outcome option).

For simplicity, let us present the procedurally stochastic maximin heuristic under the assumption of fixed procedural errors at the first step (occurring with probability ζ in both options) and valuation dependent errors as recommended above. This can be considered as a hybrid valuation and procedural error model. We assume that each of the two options consists of two outcomes each, therefore there are four possible combinations of errors in correctly ascertaining minimum and maximum values:


$$p_{A}=\{\begin{array}{ll} \frac{e^{\lambda \left(\min X_{A}\right)}}{e^{\lambda \left(\min X_{A}\right)}+e^{\lambda \left(\min X_{B}\right)}} & \text{withprob}.{\left(1-\zeta \right)}^{2}\\ \frac{e^{\lambda \left(\max X_{A}\right)}}{e^{\lambda \left(\max X_{A}\right)}+e^{\lambda \left(\min X_{B}\right)}} & \text{withprob}.\;\zeta \left(\text{1-}\zeta \right)\\ \frac{e^{\lambda \left(\min X_{A}\right)}}{e^{\lambda \left(\min X_{A}\right)}+e^{\lambda \left(\max X_{B}\right)}} & \text{withprob}.\;\zeta \left(\text{1-}\zeta \right)\\ \frac{e^{\lambda \left(\max X_{A}\right)}}{e^{\lambda \left(\max X_{A}\right)}+e^{\lambda \left(\max X_{B}\right)}} & \text{withprob}.\;\zeta^{\text{2}} \end{array}$$

A more sophisticated implementation of procedural errors for the priority heuristic can be found in Rieskamp (2008). The priority heuristic is lexicographic and considers attributes of the prospects in the following order (first to last): minimum gain, probability of minimum gain, maximum gain, and probability of maximum gain. Stopping rules, diverting to a final choice, at each step are defined by setting minimum thresholds.

1. If the minimum gains of the two prospects differ by 1/10 (or more) of the (global) maximum gain, choose the prospect with the highest minimum gain; otherwise continue to step 2.
2. If the probabilities of the minimum gains differ by 1/10 (or more) of the probability scale, choose the prospect with the highest probability of the minimum gain; otherwise continue to step 3.
3. If the maximum gains differ by 1/10 (or more) of the (global) maximum gain, choose the prospect with the highest probability of the maximum gain; otherwise continue to step 4.
4. Choose the prospect with the highest probability of the maximum gain.

Each of the steps involves a comparison between two values, which in the deterministic version occur without error. By contrast, Rieskamp (2008) assumes that the subjective difference in the two values compared at each step is a random variable with a mean equal to the real difference and non-zero variance capturing errors in the comparison. Consequently, comparing the subjective difference to the threshold of each step ultimately leads to stochastic or noisy choices. Thus, this stochastic model of the priority heuristic implements procedural errors according to our definition that are dependent on the magnitude of differences (in contrast to our maximin example above). Note that Rieskamp (2008) also estimates different threshold values, which are fixed in the deterministic version, and allows for the order of the steps to vary leading to between-participant stochasticity. However, we are here concerned with error mechanisms and stochasticity that arises within-participants.

### 3.4 Discussion

We consider procedural errors to be the most cognitively realistic error mechanism. Yet, there are disadvantages to implementing this type of mechanism relative to fixed or valuation error mechanism. The primary disadvantage is probably already apparent. It is the increase in model complexity introduced by the addition of more parameters at every processing step. The more parameters that need to be estimated, the more data are needed to identify those parameters well in the estimation and to avoid the curse of in-sample over-fitting. At some point, if too many arbitrary error parameters are introduced, this will blur the line between models of simple heuristics and flexible models. Two methodological tools may be useful in taming the problem of model complexity and identification if procedural errors are used. Instead of increasing the number of tasks in an experiment to collect more data, it may be useful to collect additional non-choice data, such as response times and process-tracing data. This data will also reduce model mimicry, as some models making similar choices may have very different implications for response times and/or information search and integration. A better understanding of the decision processes will be conducive to the addition of more appropriate procedural errors.

The advantage of the fixed and valuation mechanisms is that they can be implemented with only a single error parameter to be estimated. Let us return to our stochastic maximin example: the independent error version requires the estimation only of λ whereas the procedural version requires both λ and ζ. There is thus a tradeoff between cognitive plausibility, which we believe dictates an error mechanism at every information search or integration step (EIP) and estimation practicality. Given the constraints in the length of experiments and the number of tasks that can be reasonably presented to participants, in many cases procedural error mechanisms may not be a viable solution.

For the majority of studies, we anticipate that the most practical solution will be valuation mechanisms that implicitly aggregate the procedural errors into a single error at the valuation stage, albeit with some loss of information and misspecification of the true error distribution. Compared to fixed errors, the valuation mechanism has the advantage of being conditional on valuations, which given the existing empirical evidence cited earlier is highly likely to be relevant, and has been shown to be a significant improvement over fixed errors. At the very least, we would recommend empirical researchers to compare their deterministic heuristic to at least one stochastic version of it, following the example of Schulze et al. (2021), who actually went further by considering two stochastic versions based on fixed and valuation error mechanisms.

While researchers should decide upon which type of mechanism to employ based on the merits of each particular study and tasks, we anticipate that valuation mechanisms will often represent the best tradeoff. However, wherever possible we would encourage consideration of a simple procedural mechanism, such as the one we presented for the Maximin heuristic that only adds one more parameter. Unfortunately, for procedural models with many EIPs, the complexity and number of free parameters may quickly increase, unless all types of EIPs are assumed to have an identical error mechanism and error parameter. Even though such an assumption is not realistic, it may be a reasonable approximation and a practical solution as it avoids additional error parameters.

In general, the heuristics commonly used in the literature on decision making under risk and uncertainty are all amenable to the valuation-based error mechanism adopted in our maximin example. More specifically, any heuristic at some point must make a comparison across options. It is simple to assume that a valuation-based error mechanism operates on those values that are compared when leading to the final decision (of the deterministic heuristics). For example, for the stochastic model of the maximax heuristic, the comparison would be across the maximum values of the two options. For the stochastic model of the equiprobable heuristic, it would be the sums of all outcomes of each option. Note, that while we refer to this as a valuation stage, our suggestion remains true to the simplicity of heuristics as this “valuation” is not derived from multiplicative and probabilistic calculations (as in expected utility theory), but is simply a comparison of two values that are not transformed in any way. The approach of treating the final values that are compared by a heuristic at a decision node as a form of valuation is virtually universally applicable, and is a practical way of generating preferential strength predictions from heuristics.

This approach can also be trivially extended to lexicographic heuristics with more than one final decision node. Here the valuation error mechanism is added to whichever node makes the final decision for a specific decision problem. However, it is not clear that the same error parameter would be appropriate at each decision node, especially if the compared values are scaled very differently or even refer to very different entities. For instance, the first and third decision nodes in the priority heuristic compare outcome values, whereas the second and fourth nodes compare the outcomes' likelihoods. This is not prohibitive, but would mean that it may be necessary to estimate a different error parameter for different nodes.

## 4 Conclusion

The majority of models of choice heuristics in the literature make deterministic predictions. That is, they predict a specific choice with certainty. However, empirical evidence regarding choice stochasticity in general challenges this practice and raises important questions about whether stochastic variants of heuristics may be desirable. The few instances of stochastic heuristics, the stochastic priority model (Rieskamp, 2008) and the social circle model (Schulze et al., 2021) have confirmed the superiority of stochastic variants over their deterministic counterparts.

We have presented a simple method for converting most heuristics for choice into stochastic variants. Crucially, this technique allows heuristics to determine a strength of preference for the options under consideration, thereby allowing for errors to be conditioned on the magnitude of preferential strength. This places heuristics on a more level playing field with the flexible models using free parameters that are often used in the literature and which are typically implemented with an error mechanism that induces choice stochasticity. Stochastic variants of heuristics address the problem of misspecification when error distributions are not included and also various methodological issues that arise particularly in model comparisons. Other advantages include making heuristics more falsifiable and allowing for more informative predictive metrics that encompass probabilistic predictions instead of an all-or-nothing metric (such as % correct responses).

There is of course a tradeoff to the above, and this comes in the form of the addition of at least one free parameter to capture the magnitude of errors. While anathema to parts of the heuristic literature that reject free parameters, our proposed technique allows researchers to retain the deterministic and parameter-free core component of existing heuristics, so that the free parameters enter only through the additional error mechanism. We believe that this is an acceptable tradeoff and that the advantages will outweigh the disadvantages. Importantly, we highlighted the possibility that some existing heuristics in the literature may have been erroneously discarded as not predictive of behavior due to the fact that errors were not accounted for. Ultimately, however, the pros and cons of stochastic versions of models of heuristics should be assessed empirically and in model competitions involving flexible models, and deterministic and stochastic models of heuristics.

## Data availability statement

The original contributions presented in the study are included in the article/supplementary material, further inquiries can be directed to the corresponding author.

## Author contributions

LS: Writing – review & editing, Writing – original draft. RH: Writing – original draft, Writing – review & editing.

## Footnotes

^1^Maximax chooses the prospect with the highest maximum value, Maximin the one with the highest minimum value, Least likely the prospect with the lowest probability of the worst outcome, Most likely the prospect with the highest most-likely outcome. Equiprobable assumes that each outcome has the same probability of occurring and chooses the prospect with the highest expectation. Equiprobable eliminates outcomes whose probability are less than the inverse of the number of outcomes, and then assumes equal-weighting of the surviving outcomes to calculate a prospect's expectation. See Section 3.3 for the definition of the Priority heuristic.
